# Fixed Point Iteration Based Algorithm for Asynchronous TOA-Based Source Localization

**DOI:** 10.3390/s22186871

**Published:** 2022-09-11

**Authors:** Yanbin Zou, Jingna Fan, Liehu Wu, Huaping Liu

**Affiliations:** 1 Department of Electronic and Information Engineering, Shantou University, Shantou 515063, China; 2 School of Electrical Engineering and Computer Science, Oregon State University, Corvallis, OR 97331, USA

**Keywords:** fixed point iteration (FPI), sensor position errors, source localization, time-of-arrival (TOA)

## Abstract

This paper investigates the problem of source localization using signal time-of-arrival (TOA) measurements in the presence of unknown start transmission time. Most state-of-art methods are based on convex relaxation technologies, which possess global solution for the relaxed optimization problem. However, computational complexity of the convex optimization–based algorithm is usually large, and need CVX toolbox to solve it. Although the two stage weighted least squares (2SWLS) algorithm has very low computational complexity, its estimate performance is susceptible to sensor geometry and threshold phenomenon. A new algorithm that is directly derived from maximum likelihood estimator (MLE) is developed. The newly proposed algorithm is named as fixed point iteration (FPI); it only involves simple calculations, such as addition, multiplication, division, and square-root. Unlike state-of-the-art methods, there is no matrix inversion operation and can avoid the unstable performance incurred by singular matrix. The FPI algorithm can be easily extended to the scenario with sensor position errors. Finally, simulation results demonstrate that the proposed algorithm reaches a good balance between computational complexity and localization accuracy.

## 1. Introduction

Source localization has a wide range of applications, such as navigation, monitoring, tracking, rescue and so on. There are lots of methods can be used to determine the source position, where time-of-arrival (TOA), time-difference-of-arrival (TDOA), time-delay (TD), received signal strength (RSS), angle-of-arrival (AOA) and their combinations are commonly positioning schemes [1,2,3,4,5,6,7,8].

A TOA-based localization system requires that the source and sensors be accurately time synchronized. However, it is hard to maintain the accurate synchronization when the source is non-cooperative [9]. The pseudo ranges are corrupted with an unknown distance when the start transmission time is unknown in the asynchronous networks. As a result, study on the asynchronous TOA (ATOA)-based localization problem is very important in the practice.

In the work reported in [10,11,12,13,14,15,16,17,18], the ATOA-based localization problem can be addressed by two ways: (a) convex optimization methods, including semidefinite programming (SDP) and second-order-cone programming (SOCP) [10,11,12,13,14,15,16,17]; (b) two stage weighted least squares (2SWLS) method [18].

Convex optimization methods have the merit of global solution, but their computational complexity is usually huge and need a CVX toolbox to solve the optimization problem [19]. For example, Xu et al. [10] developed two SDP-based algorithms corresponding to two step least squares (2LS) and min–max (MMA) criterion, respectively. Then in [14], Zou et al. showed that the original 2LS-based SDP algorithm cannot provide a good solution because the weighted matrix G is singular. In order to improve the tightness of the original SDP algorithm, second-order-cone constraints and penalty term are jointly added to the original SDP algorithm. However, the algorithm proposed in [14] needs to calculate SDP problem multiple times and results in great computational burden. Recently, in [17], Ma et al. formulated an SDP algorithm to address the constraint existing in the WLS problem, which was formulated by Huang et al. in [18].

In [18], Huang et al. proposed a 2SWLS method to jointly estimate the start transmission time and the location of source. The 2SWLS method has very low computational complexity, but its localization accuracy is highly susceptible to sensor geometry when it is uniform circular array and the source is close to the array center. Besides this, the 2SWLS method presents a threshold phenomenon.

Another challenging issue for ATOA localization is sensor position errors, which could drastically degrade the accuracy [20]. Several works have addressed this problem [10,13,14,15,16], which have shown that using convex optimization could effectively address the sensor position errors. However, their computational complexity is also large.

In this paper, we develop a fixed point iteration (FPI) algorithm for the ATOA-based localization problem. The FPI algorithm is entirely and directly developed from maximum likelihood estimator (MLE). The FPI solution satisfies the equation that gradient is equal to zero. It is different from state-of-the-art methods. First, unlike weighted least squares (WLS) based algorithms, there is no approximation in its derivation. Second, unlike semidefinite programming (SDP) based algorithms, there is also no convex relaxation in its derivation. Third, unlike Gauss–Newton or Quasi-Newton based algorithms [21,22], there is no first-order Taylor series expansion in its derivation. Besides this, the proposed FPI algorithm can be easily tailored to the scenario with sensor position errors.

The rest of this paper is organized as follows. Section 2 develops an iterative algorithm when sensors positions are accurate. The algorithm is then extended to the scenario with non-accurate sensors positions in Section 3. Simulation results are presented in Section 4 to compare the performances of the proposed algorithm, state-of-art algorithms and Cramér-Rao lower bound (CRLB).

The following notations are used throughout the paper. Bold lowercase and uppercase letters denote vectors and matrices, respectively; A(:,i) denotes the *i*th column of matrix A, and A(i,:) denotes the *i*th row of matrix A; tr(A) is the trace of A; $I_{M}$ is the M×M identity matrix, $1_{M}$ is the column vector of *M* ones, and $0_{M}$ is the column vector of *M* zeros; $∥\cdot ∥$ is the $l_{2}$ norm; ⊗ is the Kroneker product of two matrices; and E[·] is the expectation.

## 2. Localization with Accurate Sensor Positions

Consider a network with *M* sensors and one source whose location $u\in R^{m\times 1}$ is unknown and to be estimated (m=2 or 3). The *i*th sensor position $s_{i}$ is accurate known.

Under line-of-sight condition, the range-of-arrival (ROA) measurements between sensor *i* and the source are expressed as

(1) $$r_{i}=t_{0}c+∥u-s_{i}∥+n_{i},\;i=1,\cdots ,M$$

where *c* is the known signal propagation speed, and $t_{0}$ is unknown start transmission time. In the next, $t_{0}c$ will be replaced by $d_{0}$, and Equation (1) can be written as

(2) $$r_{i}=d_{0}+∥u-s_{i}∥+n_{i}$$

where $n_{i},i=1,\cdots ,M,$ are the ROA measurement noise, which are modeled as zero-mean Gaussian random variables with covariance matrix $Q=\mathrm{diag}(\left[\sigma_{1}^{2},\ldots ,\sigma_{M}^{2}\right])$. The maximum likelihood estimator (MLE) from Equation (2) is formulated as

(3) $$\begin{array}{cc} \; & \min_{\begin{array}{c} u,d_{0} \end{array}}\sum_{i=1}^{M}\frac{{\left(r_{i}-d_{0}-∥u-s_{i}∥\right)}^{2}}{\sigma_{i}^{2}}. \end{array}$$

The above formulation can also be written as

(4a) $$\begin{array}{cc} \min_{\begin{array}{c} u,d_{0},d \end{array}} & {\left(r-d_{0}1_{M}-d\right)}^{T}Q^{-1}\left(r-d_{0}1_{M}-d\right) \end{array}$$

(4b) $$\begin{array}{cc} s.t. & d_{i}=∥u-s_{i}∥. \end{array}$$

where $d={\left[d_{1},\ldots ,d_{M}\right]}^{T}$. Let gradient of objective function in (4a) with respect to $d_{0}$ to zero

(5) $$\begin{array}{c} -21_{M}^{T}Q^{-1}\left(r-d_{0}1_{M}-d\right)=0 \end{array}$$

and we can obtain

(6) $$\begin{array}{c} d_{0}=\frac{1_{M}^{T}Q^{-1}\left(r-d\right)}{1_{M}^{T}Q^{-1}1_{M}}. \end{array}$$

Putting above $d_{0}$ back to Equation (4), we obtain

(7a) $$\begin{array}{cc} \min_{\begin{array}{c} u,d \end{array}} & {\left(r-d\right)}^{T}G\left(r-d\right) \end{array}$$

(7b) $$\begin{array}{cc} s.t. & d_{i}=∥u-s_{i}∥ \end{array}$$

where

(8) $$\begin{array}{c} G=I_{M}-\frac{1_{M}1_{M}^{T}Q^{-1}}{1_{M}^{T}Q^{-1}1_{M}}. \end{array}$$

Equation (7) is a non-convex optimization problem, and it can be resorted to convex relaxation method [14]. However, the convex optimization–based algorithm usually has very high computational complexity, and need CVX toolkit to solve it. Next, we develop an alternative solution for the above problem. First, Equation (7) can be expressed as the form of unconstrained optimization problem

(9) $$\begin{array}{c} \min_{\begin{array}{c} u \end{array}}\sum_{i=1}^{M}\sum_{j=1}^{M}G\left(i,j\right)\left(r_{i}-∥u-s_{i}∥\right)\left(r_{j}-∥u-s_{j}∥\right). \end{array}$$

The gradient of objective function in (9) with respect to u is

(10) $$\begin{array}{c} g=-2\sum_{i=1}^{M}\sum_{j=1}^{M}G\left(i,j\right)\left(r_{i}-∥u-s_{i}∥\right)\frac{u-s_{j}}{∥u-s_{j}∥}. \end{array}$$

The necessary condition for the optimization problem in (9) is

(11) $$\begin{array}{c} g=0 \end{array}$$

i.e.,

(12) $$\begin{array}{cc} \; & \sum_{i=1}^{M}G\left(i,i\right)\left(r_{i}\frac{u-s_{i}}{∥u-s_{i}∥}-\left(u-s_{i}\right)\right)+\sum_{i=1}^{M}\sum_{j\neq i}^{M}G\left(i,j\right)\left(r_{i}-∥u-s_{i}∥\right)\frac{u-s_{j}}{∥u-s_{j}∥}=0. \end{array}$$

Next, move the linear function of u to one side of the equation, and the constant term and nonlinear function of u to another side of the equation

(13) $$\begin{array}{cc} \; & \sum_{i=1}^{M}G\left(i,i\right)u=\sum_{i=1}^{M}G\left(i,i\right)\left(r_{i}\frac{u-s_{i}}{∥u-s_{i}∥}+s_{i}\right)+\sum_{i=1}^{M}\sum_{j\neq i}^{M}G\left(i,j\right)\left(r_{i}-∥u-s_{i}∥\right)\frac{u-s_{j}}{∥u-s_{j}∥}. \end{array}$$

Then

(14) $$\begin{array}{cc} \; & u=\frac{1}{\mathrm{tr}(G)}\left(\sum_{i=1}^{M}G\left(i,i\right)\left(r_{i}\frac{u-s_{i}}{∥u-s_{i}∥}+s_{i}\right)+\sum_{i=1}^{M}\sum_{j\neq i}^{M}G\left(i,j\right)\left(r_{i}-∥u-s_{i}∥\right)\frac{u-s_{j}}{∥u-s_{j}∥}\right). \end{array}$$

Moreover,

(15) $$\begin{array}{c} \mathrm{tr}\left(G\right)=\mathrm{tr}\left(I_{M}-\frac{1_{M}1_{M}^{T}Q^{-1}}{1_{M}^{T}Q^{-1}1_{M}}\right)=M-1. \end{array}$$

As a result, the FPI computation of u is

(16) $$\begin{array}{cc} \; & u_{k}=f\left(u_{k-1}\right)=\frac{1}{M-1}\left(\sum_{i=1}^{M}G\left(i,i\right)\left(r_{i}\frac{u_{k-1}-s_{i}}{∥u_{k-1}-s_{i}∥}+s_{i}\right)+\sum_{i=1}^{M}\sum_{j\neq i}^{M}G\left(i,j\right)\left(r_{i}-∥u_{k-1}-s_{i}∥\right)\frac{u_{k-1}-s_{j}}{∥u_{k-1}-s_{j}∥}\right). \end{array}$$

It should be noted that the above derivations are based on the necessary condition. In other words, the result from above FPI may be a stationary point, i.e., a local maximum, a local minimum point or a saddle point. In order to obtain the global minimum point, we adopt the following steps. First is to use multiple initial values to calculate FPI in Equation (16). Without loss of generality, the set of initial values can be set as follows (from (16), it can be seen that the initial values should be not equal to the location of sensors. The main principle for the setting of initial values is that it includes points located inside and outside the convex hull of sensors).

(17) $$\begin{array}{c} U_{0}=\left[\frac{1}{M}\sum_{i=1}^{M}s_{i},2s_{1}-\frac{1}{M}\sum_{i=1}^{M}s_{i},\ldots ,2s_{M}-\frac{1}{M}\sum_{i=1}^{M}s_{i}\right]. \end{array}$$

Then, we calculate the corresponding cost function for each initial value

(18) $$\begin{array}{c} c_{f}\left(n\right)={\left(r-d_{n}^{*}\right)}^{T}G\left(r-d_{n}^{*}\right),n=1,\ldots ,M+1 \end{array}$$

where $d_{n}^{*}={\left[∥u_{n}^{*}-s_{1}∥,\ldots ,∥u_{n}^{*}-s_{M}∥\right]}^{T}$. Finally, we choose the result that minimizes the cost function.

Besides this, in order to reduce the calculation amount, we take the following measure. If $c_{f}\left(n\right)<\mu$ for a certain *n*, then we stop calculating the iterations for the rest initial values. Next, we show how to determine the threshold μ. Reviewing (3), we can obtain

(19) $$\begin{array}{c} E\left[\sum_{i=1}^{M}\frac{{\left(r_{i}-d_{0}-∥u-s_{i}∥\right)}^{2}}{\sigma_{i}^{2}}\right]=E\left[\sum_{i=1}^{M}\frac{n_{i}^{2}}{\sigma_{i}^{2}}\right]=M \end{array}$$

and

(20) $$\begin{array}{c} E[{\left(r-d\right)}^{T}G\left(r-d\right)]=M \end{array}$$

for true source location u. As a result, the threshold is set to μ=M.

The above proposed algorithm procedures are summarized in Algorithm 1.
**Algorithm 1:** Localization with scenario 1: accurate sensor position.
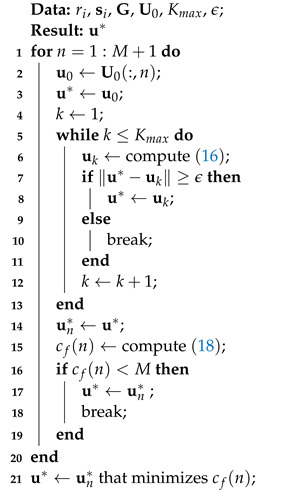


## 3. Localization with Non-Accurate Sensor Positions

In this section, we will extend the FPI localization algorithm to the scenario with non-accurate sensor positions. First, the *i*th true but unknown sensor location is $s_{i}^{0}$. The available sensor location can be denoted as

(21) $$s_{i}=s_{i}^{0}+\beta_{i},\;i=1,\cdots ,M$$

where $\beta_{i}$ is a zero-mean white Gaussian vector with covariance matrix $\delta_{i}^{2}I_{m}$ [23]. The MLE is formulated as

(22) $$\begin{array}{cc} \; & \min_{\begin{array}{c} u,d_{0},s_{i}^{0} \end{array}}\sum_{i=1}^{M}\frac{{\left(r_{i}-d_{0}-∥u-s_{i}^{0}∥\right)}^{2}}{\sigma_{i}^{2}}+\sum_{i=1}^{M}\frac{∥s_{i}-s_{i}^{0}∥}{\delta_{i}^{2}}. \end{array}$$

Putting (6) into (22), this results in

(23a) $$\begin{array}{cc} \min_{\begin{array}{c} u,d,s_{i}^{0} \end{array}} & {\left(r-d\right)}^{T}G\left(r-d\right)+\sum_{i=1}^{M}\frac{{∥s_{i}-s_{i}^{0}∥}^{2}}{\delta_{i}^{2}} \end{array}$$

(23b) $$\begin{array}{cc} s.t. & d_{i}=∥u-s_{i}^{0}∥. \end{array}$$

Let $x=[u;s_{1}^{0};\ldots ;s_{M}^{0}]$, $s=[s_{1};\ldots ;s_{M}]$, $W_{s}=Q_{s}⊗I_{m}$, $Q_{s}=\mathrm{diag}\left(\left[\frac{1}{\delta_{1}^{2}},\ldots ,\frac{1}{\delta_{M}^{2}}\right]\right)$, $P_{i}=\left[I_{m},0_{m,m(i-1)},-I_{m},0_{m,m(M-i)}\right]$, $B=[0_{mM,m},I_{mM}]$. Equation (23) can be written as

(24) $$\begin{array}{cc} \min_{\begin{array}{c} x \end{array}}\; & \sum_{i=1}^{M}\sum_{j=1}^{M}G\left(i,j\right)\left(r_{i}-∥P_{i}x∥\right)\left(r_{j}-∥P_{j}x∥\right)+\sum_{i=1}^{mM}W_{s}\left(i,i\right){\left(s(i)-B(i,:)x\right)}^{2}. \end{array}$$

The gradient of objective function in Equation (24) with respect to x is

(25) $$\begin{array}{cc} g= & -2\sum_{i=1}^{M}\sum_{j=1}^{M}G\left(i,j\right)\left(r_{i}-∥P_{i}x∥\right)\frac{P_{j}^{T}P_{j}x}{∥P_{j}x∥}-2\sum_{i=1}^{mM}W_{s}\left(i,i\right)\left(s(i)-B(i,:)x\right)B{\left(i,:\right)}^{T}. \end{array}$$

The necessary condition for the optimization problem in (24) is

(26) $$\begin{array}{c} g=0. \end{array}$$

Equivalent to

(27) $$\begin{array}{cc} \; & \sum_{i=1}^{M}G\left(i,i\right)r_{i}\frac{P_{i}^{T}P_{i}x}{∥P_{i}x∥}-\sum_{i=1}^{M}G\left(i,i\right)P_{i}^{T}P_{i}x+\sum_{i=1}^{M}\sum_{j\neq i}^{M}G\left(i,j\right)\left(r_{i}-∥P_{i}x∥\right)\frac{P_{j}^{T}P_{j}x}{∥P_{j}x∥}+\\ & \sum_{i=1}^{mM}W_{s}\left(i,i\right)s\left(i\right)B{\left(i,:\right)}^{T}-\sum_{i=1}^{mM}W_{s}\left(i,i\right)B{\left(i,:\right)}^{T}B\left(i,:\right)x=0 \end{array}$$

i.e.,

(28) $$\begin{array}{cc} \; & \left(\sum_{i=1}^{M}G\left(i,i\right)P_{i}^{T}P_{i}+\sum_{i=1}^{mM}W_{s}\left(i,i\right)B{\left(i,:\right)}^{T}B\left(i,:\right)\right)x=\sum_{i=1}^{M}G\left(i,i\right)r_{i}\frac{P_{i}^{T}P_{i}x}{∥P_{i}x∥}+\sum_{i=1}^{M}\sum_{j\neq i}^{M}G\left(i,j\right)\left(r_{i}-∥P_{i}x∥\right)\frac{P_{j}^{T}P_{j}x}{∥P_{j}x∥}\\ \; & +\sum_{i=1}^{mM}W_{s}\left(i,i\right)s\left(i\right)B{\left(i,:\right)}^{T}. \end{array}$$

Finally,

(29) $$\begin{array}{cc} \; & x={\left(\sum_{i=1}^{M}G\left(i,i\right)P_{i}^{T}P_{i}+\sum_{i=1}^{mM}W_{s}\left(i,i\right)B{\left(i,:\right)}^{T}B\left(i,:\right)\right)}^{-1}\cdot \\ \; & [\sum_{i=1}^{M}G\left(i,i\right)r_{i}\frac{P_{i}^{T}P_{i}x}{∥P_{i}x∥}+\sum_{i=1}^{M}\sum_{j\neq i}^{M}G\left(i,j\right)\left(r_{i}-∥P_{i}x∥\right)\frac{P_{j}^{T}P_{j}x}{∥P_{j}x∥}+\sum_{i=1}^{mM}W_{s}\left(i,i\right)s\left(i\right)B{\left(i,:\right)}^{T}]. \end{array}$$

The FPI computation of x is

(30) $$\begin{array}{cc} \; & x_{k}={\left(\sum_{i=1}^{M}G\left(i,i\right)P_{i}^{T}P_{i}+\sum_{i=1}^{mM}W_{s}\left(i,i\right)B{\left(i,:\right)}^{T}B\left(i,:\right)\right)}^{-1}\cdot \\ \; & [\sum_{i=1}^{M}G\left(i,i\right)r_{i}\frac{P_{i}^{T}P_{i}x_{k-1}}{∥P_{i}x_{k-1}∥}+\sum_{i=1}^{mM}W_{s}\left(i,i\right)s\left(i\right)B{\left(i,:\right)}^{T}+\sum_{i=1}^{M}\sum_{j\neq i}^{M}G\left(i,j\right)\left(r_{i}-∥P_{i}x_{k-1}∥\right)\frac{P_{j}^{T}P_{j}x_{k-1}}{∥P_{j}x_{k-1}∥}]. \end{array}$$

The proposed algorithm for scenario 2 is shown in Algorithm 2. It includes two stages. The first stage is to carry out Algorithm 1 to obtain an initial guess of source position by using the non-accurate sensor positions. The second stage is to update x using Equation (30) and taking the result from stage one as initial value.
**Algorithm 2:** Localization with scenario 2: non-accurate sensor positions.
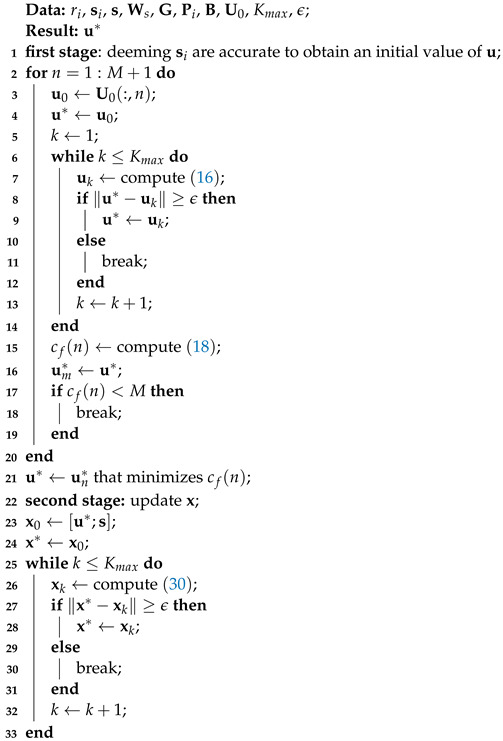


## 4. Simulation Results

In this section, two scenarios with accurate and non-accurate sensor positions are respectively considered to demonstrate the performance of two proposed algorithms.

In the first scenario, we compare several algorithms: the proposed Algorithm 1 (with one middle point as initial value labeled as ‘Proposed-Mid’, with multiple initial values labeled as ‘Proposed-Multi’), the Gauss–Newton method (in the simulation, the result of Gauss–Newton method may be NaN due to the matrix is singular in the calculation of matrix inversion. In order to avoid this case, it is set that, if the distance between the updated estimate and the initial value is large than 1000, then the iteration will be stopped and output the initial value as its result) with one middle point as initial value labeled as ‘Gauss–Newton-Mid’, with multiple initial values labeled as ‘Gauss–Newton-Multi’; the derivation for the Gauss–Newton method is shown in Appendix A), 2SWLS [18], SDP-Zou [14], SDP–Ma [17] and CRLB.

In the second scenario, we compare the proposed Algorithm 2 (labeled as ‘Proposed’) with the Gauss–Newton method (labeled as ‘Gauss–Newton’), SDP–Zou [14] and CRLB when sensor positions are non-accurate. The derivation of the Gauss–Newton method with position errors for the problem of (24) is given in Appendix B.

The setting of parameters are the same as in [14]: a network with four sensors, and one source is simulated. The positions of the sensors are ${\left[-10,-10\right]}^{T}m$, ${\left[-10,10\right]}^{T}m$, ${\left[10,-10\right]}^{T}m$, ${\left[10,10\right]}^{T}m$, and the location of source is randomly chosen from a square [−15,15]m×[−15,15]m. The start transmission time $t_{0}$ is drawn from uniform distribution U[10,40]ns. Sensors’ position covariance matrix is $\delta^{2}I_{2}$. The range measurement covariance matrix is $Q=\sigma^{2}I_{M}$. The initial set for Algorithm 1 is $U_{0}=[0,-20,-20,20,20;\;0,-20,20,$−20,20]. Five penalty factors $\eta_{1},\eta_{2},\eta_{3},\eta_{4},\eta_{5}$ equal to $10^{-4}$, $10^{-3}$, $10^{-2}$, $10^{-1}$, $10^{0}$ are used for the SDP–Zou algorithm. The SDP–Zou and SDP–Ma algorithms are implemented by CVX toolbox using SeDuMi as a solver and with best precision [24]. The Gauss–Newton methods and the proposed FPI algorithm have same initials. Root mean square errors (RMSEs) are drawn from 4000 Monte Carlo realizations in the following simulations.

**Figure 1:**
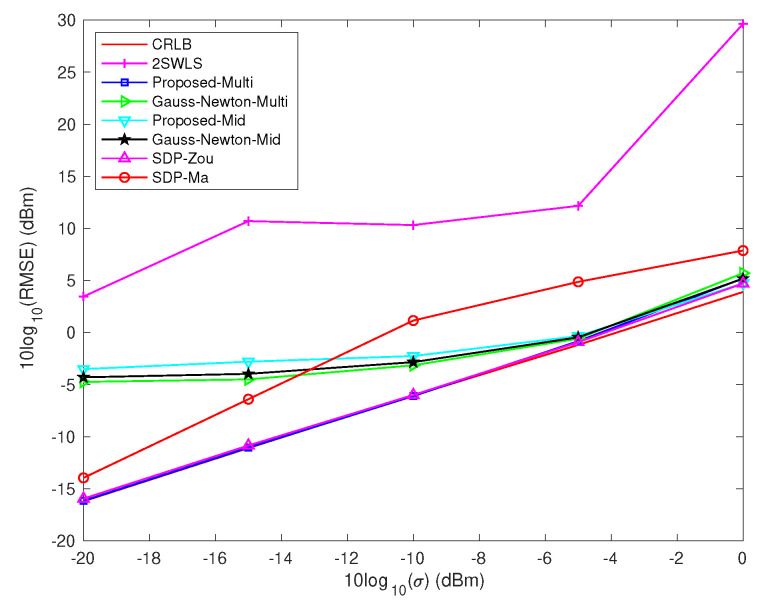
RMSE vs. σ in scenario 1.

From Figure 1, we have following observations: (1) only the proposed algorithm with multiple initial values and the SDP–Zou algorithm can reach CRLB; (2) the ‘Proposed-Multi’ algorithm is superior to the ‘Proposed-Mid’ algorithm, which validates the existence of other stationary point except for the global minimum point in the problem of MLE; (3) the Gauss–Newton methods have inferior performance due to its first-order approximation in the derivations; (4) the 2SWLS algorithm has the worst performance even with small noise condition, which is due to the sensor geometry is uniform circular array and the source is close to the array center occasionally [25]; (5) the SDP–Ma algorithm has poor performance, which is due to the number of sensors, namely, 4, which results in rank-1 solution being hard to obtain [17].

Besides this, the average running times of different algorithms are given in Table 1. It can be seen that: (1) the 2SWLS algorithm has the lowest computation time, because it only involves three times computation of WLS solution; (2) both SDP–Zou and SDP–Ma algorithms are time-consuming to obtain the solutions by CVX toolbox; moreover, the SDP–Zou algorithm needs to solve the SDP problem five times; (3) the average running time of ‘Proposed-Multi’ is large than but less than twice of the ‘Proposed-Mid’, which validates that the stop criterion in (20) can help to reduce the calculation amount. In the simulation, we found that if μ=2M the calculation amount can be further reduced; (4) the Gauss–Newton algorithms are faster than the proposed FPI algorithms.

**Figure 2:**
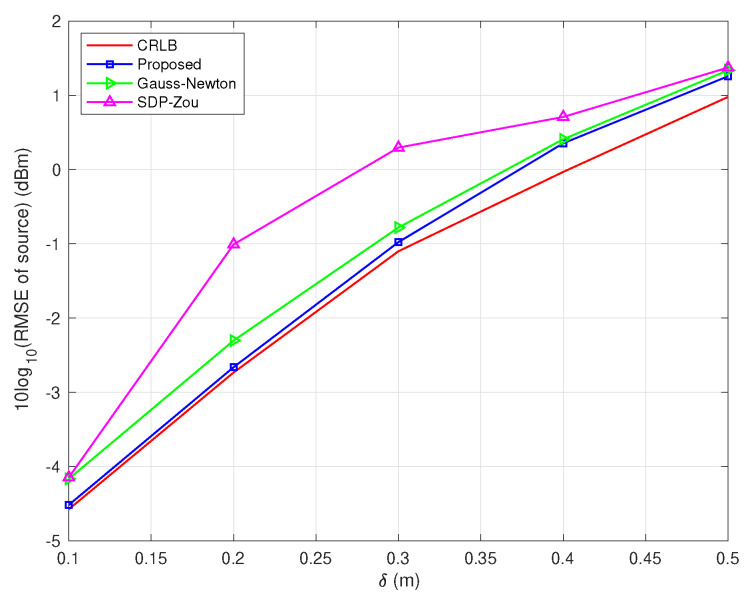
RMSE of source vs. δ with σ=0.1 m in scenario 2.

**Figure 3:**
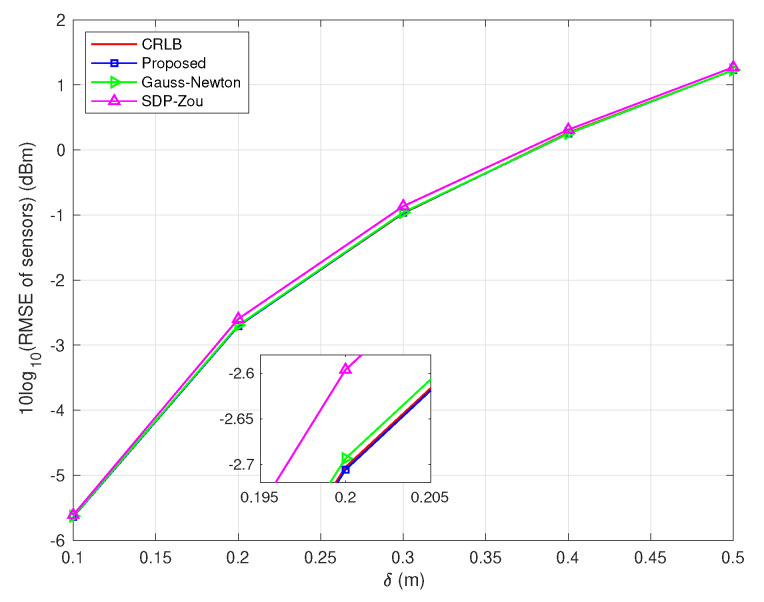
RMSE of sensors vs. δ with σ=0.1 m in scenario 2.

Figure 2 and Figure 3, respectively, evaluate the source and sensors estimate performances of different algorithms versus δ when the locations of sensors are non-accurate. From these two figures, it is observed that the performance of the proposed algorithm is superior to the other two algorithms. Besides this, in Table 1, it can be seen that the average running time of the proposed algorithm is much less than the SDP–Zou algorithm, but greater than the Gauss–Newton algorithm.

Finally, from the above results and analyses, we can obtain a conclusion: The proposed FPI algorithms reach a good balance between estimation accuracy and calculation amount.

## 5. Conclusions

Two FPI algorithms for ATOA-based localization problem are respectively developed corresponding to the scenarios with accurate and non-accurate sensor positions. First, the problem of MLE for the unknown start transmission time and source position is formulated. Next, the gradient of objective function in MLE is derived, then we simplify the equation with gradient equal to zero. Finally, we extract the linear variable to one side of the equation and obtain the FPI algorithm. The simulation results validate the performances of the two proposed algorithms in terms of accuracy and computation.

## Figures and Tables

**Table 1:** The average running time [ms] of the considered algorithms with the two scenarios, in which a star denotes that the algorithm is not fitting for a certain scenario. CPU: i7-8700 3.2 GHz.

	Scenarios	1	2
Algorithms			
2SWLS		0.19	*
Proposed-Multi		8.6	*
Gauss–Newton-Multi		0.94	*
Proposed-Mid		5.3	*
Gauss–Newton-Mid		0.67	*
SDP–Ma		412	*
SDP–Zou		1773	1861
Proposed		*	28.6
Gauss–Newton		*	2.6

## Data Availability

Not applicable.

## Sample Availability

The codes of simulations are available from the authors via emails ybzou@stu.edu.cn and huaping.liu@oregonstate.edu.

## Abbreviations

The following abbreviations are used in this manuscript:

TOA	Time-of-arrival
2SWLS	Two-step weighted least squares
MLE	Maximum likelihood
PFI	Fixed point iteration
TDOA	Time-difference-of-arrival
TD	Time delay
RSS	Received signal strength
AOA	angle-of-arrival
ATOA	Asynchronous TOA
SDP	Semidefinite programming
SOCP	Second-order cone programming
WLS	Weighted least squares
CRLB	Cramer-Rao lower bound
RMSE	Root mean-square error

## Appendix A. Gauss–Newton Method for Scenario 1

In this appendix, the Gauss–Newton method for scenario 1 with accurate sensor positions is derived. Rewriting the MLE problem in (9)

(A1) $$\begin{array}{c} \min_{\begin{array}{c} u \end{array}}\sum_{i=1}^{M}\sum_{j=1}^{M}G\left(i,j\right)\left(r_{i}-∥u-s_{i}∥\right)\left(r_{j}-∥u-s_{j}∥\right) \end{array}$$

The first-order Taylor series expansion of $∥u-s_{i}∥$ is

(A2) $$\begin{array}{c} ∥u-s_{i}∥\approx ∥u_{0}-s_{i}∥+\frac{{\left(u_{0}-s_{i}\right)}^{T}\left(u-u_{0}\right)}{∥u_{0}-s_{i}∥} \end{array}$$

Then, taking (A2) into (A1) results

(A3) $$\begin{array}{cc} \min_{\begin{array}{c} u \end{array}}\sum_{i=1}^{M}\sum_{j=1}^{M} & G\left(i,j\right)\left(r_{i}-∥u_{0}-s_{i}∥-\frac{{\left(u_{0}-s_{i}\right)}^{T}\left(u-u_{0}\right)}{∥u_{0}-s_{i}∥}\right)\left(r_{j}-∥u_{0}-s_{j}∥-\frac{{\left(u_{0}-s_{j}\right)}^{T}\left(u-u_{0}\right)}{∥u_{0}-s_{j}∥}\right) \end{array}$$

Let the gradient of objective function in (A3) with u to zero

(A4) $$\begin{array}{cc} \sum_{i=1}^{M}\sum_{j=1}^{M} & -2G\left(i,j\right)\left(r_{i}-∥u_{0}-s_{i}∥-\frac{{\left(u_{0}-s_{i}\right)}^{T}\left(u-u_{0}\right)}{∥u_{0}-s_{i}∥}\right)\frac{\left(u_{0}-s_{j}\right)}{∥u_{0}-s_{j}∥}=0 \end{array}$$

Set

(A5a) $$\begin{array}{cc} \; & h_{0}={\left[r_{1}-∥u_{0}-s_{1}∥,\ldots ,r_{M}-∥u_{0}-s_{M}∥\right]}^{T} \end{array}$$

(A5b) $$\begin{array}{cc} \; & H_{0}=\left[\frac{\left(u_{0}-s_{1}\right)}{∥u_{0}-s_{1}∥},\ldots ,\frac{\left(u_{0}-s_{M}\right)}{∥u_{0}-s_{M}∥}\right] \end{array}$$

Then (A4) can be recast as

(A6) $$\begin{array}{c} H_{0}Gh_{0}=H_{0}GH_{0}^{T}\left(u-u_{0}\right) \end{array}$$

Finally, the updating of u is

(A7) $$\begin{array}{c} u=u_{0}+{\left(H_{0}GH_{0}^{T}\right)}^{-1}H_{0}Gh_{0} \end{array}$$

## Appendix B. Gauss–Newton Method for Scenario 2

In this appendix, the Gauss–Newton method for scenario 2 with non-accurate sensor positions is derived. Rewriting the MLE problem in (24)

(A8) $$\begin{array}{cc} \min_{\begin{array}{c} x \end{array}}\; & \sum_{i=1}^{M}\sum_{j=1}^{M}G\left(i,j\right)\left(r_{i}-∥P_{i}x∥\right)\left(r_{j}-∥P_{j}x∥\right)+\sum_{i=1}^{mM}W_{s}\left(i,i\right){\left(s(i)-B(i,:)x\right)}^{2} \end{array}$$

The first-order Taylor series expansion of $∥P_{i}x∥$ is

(A9) $$\begin{array}{c} ∥P_{i}x∥\approx ∥P_{i}x_{0}∥+\frac{{\left(P_{i}^{T}P_{i}x_{0}\right)}^{T}\left(x-x_{0}\right)}{∥P_{i}x_{0}∥} \end{array}$$

Then, taking (A9) into (A8) results

(A10) $$\begin{array}{cc} \min_{\begin{array}{c} u \end{array}}\; & \sum_{i=1}^{M}\sum_{j=1}^{M}G\left(i,j\right)\left(r_{i}-∥P_{i}x_{0}∥-\frac{{\left(P_{i}^{T}P_{i}x_{0}\right)}^{T}\left(x-x_{0}\right)}{∥P_{i}x_{0}∥}\right)\left(r_{j}-∥P_{j}x_{0}∥-\frac{{\left(P_{j}^{T}P_{j}x_{0}\right)}^{T}\left(x-x_{0}\right)}{∥P_{j}x_{0}∥}\right)+\\ \; & \sum_{i=1}^{mM}W_{s}\left(i,i\right){\left(s(i)-B(i,:)x\right)}^{2} \end{array}$$

Let the gradient of objective function in (A10) with x to zero

(A11) $$\begin{array}{cc} \; & \sum_{i=1}^{M}\sum_{j=1}^{M}-2G\left(i,j\right)\left(r_{i}-∥P_{i}x_{0}∥-\frac{{\left(P_{i}^{T}P_{i}x_{0}\right)}^{T}\left(x-x_{0}\right)}{∥P_{i}x_{0}∥}\right)\frac{P_{j}^{T}P_{j}x_{0}}{∥P_{j}x_{0}∥}-\sum_{i=1}^{mM}2W_{s}\left(i,i\right)\left(s(i)-B(i,:)x\right)B{\left(i,:\right)}^{T}=0 \end{array}$$

Set

(A12a) $$\begin{array}{cc} \; & h_{0}={\left[r_{1}-∥P_{1}x_{0}∥,\ldots ,r_{M}-∥P_{M}x_{0}∥\right]}^{T} \end{array}$$

(A12b) $$\begin{array}{cc} \; & H_{0}=\left[\frac{P_{1}^{T}P_{1}x_{0}}{∥P_{1}x_{0}∥},\ldots ,\frac{P_{M}^{T}P_{M}x_{0}}{∥P_{M}x_{0}∥}\right] \end{array}$$

Then (A11) can be recast as

(A13) $$\begin{array}{c} H_{0}G\left(h_{0}-H_{0}^{T}\left(x-x_{0}\right)\right)+B^{T}W_{s}\left(s-Bx\right)=0 \end{array}$$

Finally, the updating of x is

(A14) $$\begin{array}{c} x={\left(H_{0}GH_{0}^{T}+B^{T}W_{s}B\right)}^{-1}\left(H_{0}G\left(h_{0}+H_{0}^{T}x_{0}\right)+B^{T}W_{s}s\right) \end{array}$$
